# Giant Photoluminescence Enhancement and Carrier Dynamics in MoS_2_ Bilayers with Anomalous Interlayer Coupling

**DOI:** 10.3390/nano11081994

**Published:** 2021-08-03

**Authors:** Han Li, Yating Ma, Zhongjie Xu, Xiang’ai Cheng, Tian Jiang

**Affiliations:** 1 College of Advanced Interdisciplinary Studies, National University of Defense Technology, Changsha 410073, China; kadurjie@163.com (H.L.); yating_martina@163.com (Y.M.); xzj0243@163.com (Z.X.); xiang_ai_cheng@126.com (X.C.); 2 Beijing Institute for Advanced Study, National University of Defense Technology, Beijing 100000, China

**Keywords:** molybdenum disulfide, interlayer coupling, photoluminescence, pump-probe

## Abstract

Fundamental researches and explorations based on transition metal dichalcogenides (TMDCs) mainly focus on their monolayer counterparts, where optical densities are limited owing to the atomic monolayer thickness. Photoluminescence (PL) yield in bilayer TMDCs is much suppressed owing to indirect-bandgap properties. Here, optical properties are explored in artificially twisted bilayers of molybdenum disulfide (MoS_2_). Anomalous interlayer coupling and resultant giant PL enhancement are firstly observed in MoS_2_ bilayers, related to the suspension of the top layer material and independent of twisted angle. Moreover, carrier dynamics in MoS_2_ bilayers with anomalous interlayer coupling are revealed with pump-probe measurements, and the secondary rising behavior in pump-probe signal of B-exciton resonance, originating from valley depolarization of A-exciton, is firstly reported and discussed in this work. These results lay the groundwork for future advancement and applications beyond TMDCs monolayers.

## 1. Introduction

Transition metal dichalcogenides (TMDCs) have been widely viewed as promising candidates in the next generation of nanophotonics [1,2,3,4,5,6,7,8,9], electronics [10,11,12,13,14,15] and valleytronics [16,17,18,19,20,21,22,23] due to their unique optical properties and spin-valley configurations. As characterized by interlayer van der Waal forces [24], optical properties of two-dimensional TMDCs are dominated by excitons and vary significantly with their layer index. While monolayer TMDCs, such as molybdenum disulfide (MoS_2_), are direct bandgap materials with evident emission in the visible range, bilayer and few-layer TMDCs turn to be indirect-bandgap semiconductors with much suppressed photoluminescence (PL) yield [10,25,26,27,28,29]. As a result, fundamental researches of physics and explorations of functional devices based on TMDCs mainly focus on their monolayer counterparts [10], where optical densities are limited with atomic monolayer thickness [30,31]. In order to utilize the optical densities with increasing layer thicknesses, various approaches have been developed to enhance the PL yield of bilayer and few-layer TMDCs such as applying strain [32,33,34] or lateral electric fields [35], modifying growth conditions [30], and intercalation of light atomic species in the interlayer gap [31]. While these methods help increase the PL intensity of bilayer and few-layer TMDCs to some extent, there are also some realistic limitations accompanying them such as limited enhancement of emission and formation of the localized excitonic state. In particular, giant enhancement of PL intensity in bilayer TMDCs, exceeding that of monolayer counterparts, has not been reported yet.

In this work, both optical properties and carrier dynamics are explored in artificially twisted MoS_2_ bilayers. While most twisted bilayers exhibit expected normal interlayer coupling with twisted angles, unexpected anomalous interlayer coupling is firstly observed in other MoS_2_ bilayers, resulting in giant PL enhancement and blueshifted emission peaks that are related to suspension of top layer material and independent of the twisted angles. The carrier dynamics in MoS_2_ bilayers with anomalous interlayer coupling are revealed with pump-probe measurements. Moreover, the secondary rising behavior in pump-probe signal of B-exciton resonance is firstly reported and analyzed, which originates from valley depolarization of A-exciton. Based on the observed experimental results, a corresponding phenomenological model of carrier dynamics is therefore established. These results can help recognize the intrinsic optical properties of TMDCs with interlayer coupling and shed light on the potential applications beyond TMDCs monolayers.

## 2. Materials and Methods

### 2.1. Sample Preparation

Twisted MoS_2_ bilayers were prepared by a widely used wet-transfer method. A piece of MoS_2_ monolayer sample, grown on SiO_2_ substrate from chemical vapor deposition (CVD), was placed on a heating stage and then coated with one drop of anisole with 4% polymethylmethacrylate (PMMA). Afterwards, the heating stage was set to 100 °C to obtain an uneven PMMA film, which was then corroded in 2 mol/L KOH solution for 2 h and bathed in deionized water three times. Then, the PMMA film was transferred onto a clean SiO_2_ wafer to dry naturally and baked at 80 °C for 3 min. After that, the MoS_2_ sample with PMMA layer was soaked in acetone three times, for 30 min each time. In this way, the bottom layer of MoS_2_ was prepared. Likewise, the top layer of MoS_2_, from another piece of CVD-grown sample, was then transferred onto the as-prepared bottom layer sample to form numerous twisted bilayers. Each time after dissolving the PMMA layer in acetone, the MoS_2_ sample was annealed in Ar atmosphere at 400 °C for 3 h to remove the residual.

**Figure 1:**
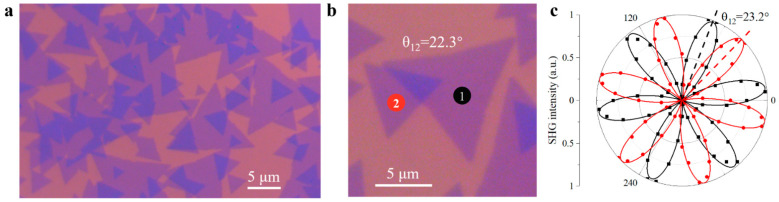
Characterization of twisted angle of MoS_2_ bilayers. (**a**) Optical image of twisted MoS_2_ bilayers. (**b**) Twisted angle of a bilayer sample, measured from optical image. (**c**) Twisted angle of bilayer sample in (**b**), determined by SHG measurements.

The optical image of fabricated twisted MoS_2_ bilayers is shown in Figure 1a, with darker color representing bilayer regions. Twisted angle of fabricated bilayers was directly determined by corresponding optical image and then confirmed from the second-harmonic-generation (SHG) measurements [19,36,37,38], as can be seen in Figure 1b,c. The two consistent results show a deviation less than 1°.

### 2.2. PL, Raman and Pump-Probe Measurements

The PL and Raman measurements were conducted with a lab-built confocal system. A 532 nm continuous-wave laser was utilized as excitation during PL mapping and Raman measurements. For polarization-resolved PL measurements, a quarter-wave plate and a Glan polarizer with horizonal polarization were set in front of the spectrometer. The orthogonally circularly polarized PL signals were detected by rotating the quarter-wave plate. The pump-probe system was based on a Ti: sapphire laser (1 kHz, Spectra-Physics), which was described in detail in a previous report [39]. The main power of the Ti: sapphire laser was sent to pump an optical parametric amplifier, generating tunable output energy as pump beam. A small portion of the femtosecond laser was sent through a sapphire crystal to generate supercontinuum white light, ranging from 470 to 1100 nm, as a probe beam. Limited by the experimental conditions, both the pump and probe beams were linearly polarized. All measurements were carried out at room temperature.

## 3. Results and Discussion

### 3.1. Optical Properties of MoS_2_ Bilayers with Normal Interlayer Coupling

**Figure 2:**
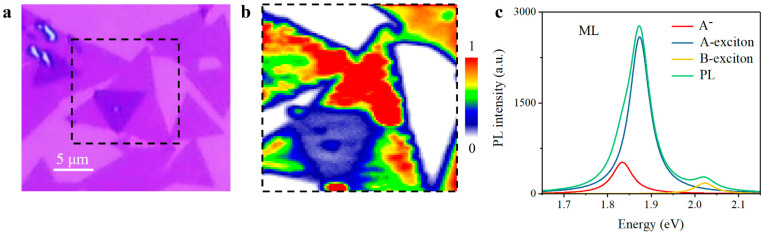
PL mapping of a twisted MoS_2_ bilayer. (**a**) Optical image. (**b**) PL mapping result of region marked in (**a**). PL intensity is integrated within A-exciton resonance. (**c**) PL spectrum of monolayer MoS_2_. ML, monolayer; A^-^, trion.

**Figure 3:**
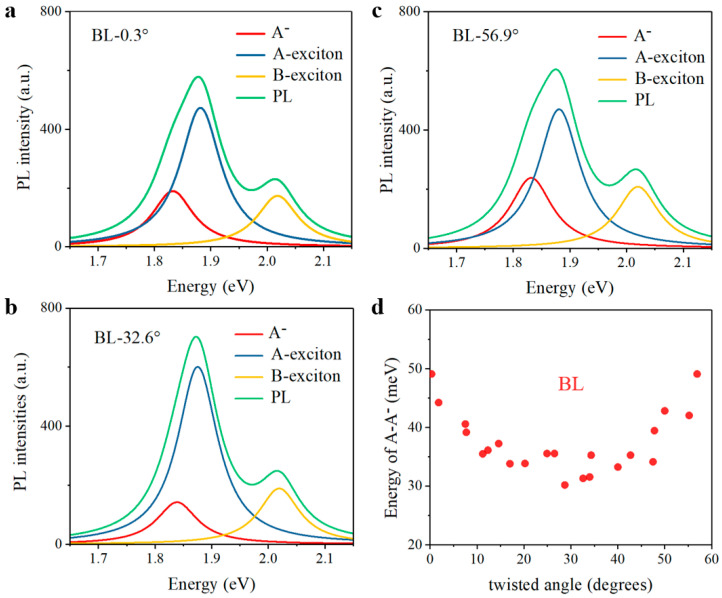
PL spectra of twisted MoS_2_ bilayers. (**a**–**c**) PL spectra of (**a**) 0.3°, (**b**) 32.6° and (**c**) 56.9° twisted MoS_2_ bilayers. (**d**) Trion binding energies collected in different twisted bilayers from 0° to 60°. BL, normal bilayer; A^-^, trion.

TMDCs monolayers are direct-bandgap semiconductors, while they turn to be indirect-bandgap materials with larger layer thickness as a result of interlayer coupling, resulting in lower PL yield [36,37,40]. After the sample preparation, the PL mapping measurement was conducted for a prototypical twisted MoS_2_ bilayer under excitation of a 532 nm continuous-wave laser, with PL intensity integrated within A-exciton resonance. As expected, PL intensity decreased evidently in the bilayer region, indicating the indirect-bandgap property in bilayer region owes to efficient interlayer coupling, which can be seen in Figure 2. To more fully understand the optical properties of twisted MoS_2_ bilayers with interlayer coupling, we then carried out PL and Raman measurements for MoS_2_ bilayers with different twisted angles, ranging from 0° to 60°, owing to the intrinsic three-fold symmetry of MoS_2_ monolayers. We firstly performed the PL measurements of twisted MoS_2_ bilayers, and the prototypical PL spectra of three twisted MoS_2_ bilayers, corresponding to 0.3° (near-0°), 32.6° (near-30°), and 56.9° (near-60°), are illustrated in Figure 3a–c, respectively. These PL spectra were fitted with a Lorentz model including trion (A^-^), A-exciton, and B-exciton for comparison, and the emission peaks of A-exciton and B-exciton in bilayers barely shift, compared with the prototypical PL spectrum of monolayer MoS_2_ in Figure 2c. However, the variation of trion peaks with twisted angles is evident. Trion peaks of 0.3° and 56.9° bilayers are redshifted compared with that of the 32.6° bilayer. For a deeper understanding, the difference of emission peaks between A-exciton and trion, usually referring to trion binding energy [40,41], was collected, varying with twisted angle in Figure 3d. Specifically, trion binding energy maximizes in near-0° and near-60° bilayers and minimizes in near-30° bilayers, which is consistent with higher emission ratio of trion in PL spectra in Figure 3a,c, also agrees well with previous studies of twisted MoS_2_ bilayers [40]. Different from tightly bound excitons with giant binding energy in TMDCs, trions have much less binding energy [42,43,44], and hence are more likely to be influenced and modulated by interlayer coupling strength. The twisted-angle-dependent variation of trion binding energy implied that the interlayer coupling strength is related to the twisted angle of the bilayers.

**Figure 4:**
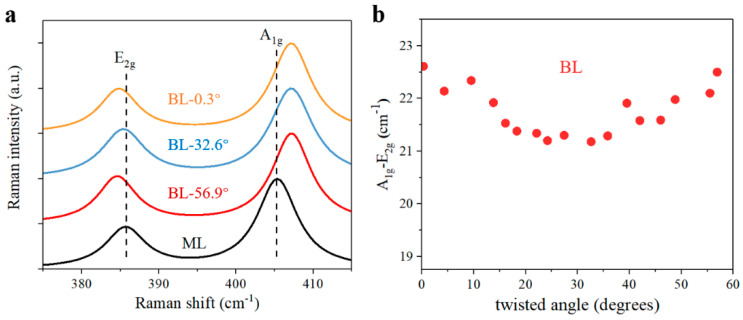
Raman spectra of twisted MoS_2_ bilayers. (**a**) Raman signals of out-of-plane $A_{1g}$ and $E_{2g}$ in-plane vibration modes in prototypical monolayer and twisted bilayers. ML, monolayer. (**b**) Raman interval of $A_{1g}$ and $E_{2g}$ vibration modes. BL, normal bilayer.

Apart from the modulated PL spectra, we then also performed Raman measurements with a 532 nm continuous-wave laser, which help verify interlayer coupling strength in MoS_2_ bilayers [36,37,40], as shown in Figure 4a. As for the Raman signals, the out-of-plane $A_{1g}$ and in-plane $E_{2g}$ vibration modes are most concerned, which can reflect the interlayer distance and resultant interlayer coupling strength. In Figure 4b, the Raman interval of $A_{1g}$ and $E_{2g}$ peaks exhibits obvious twisted-angle-dependent trend in MoS_2_ bilayers, as expected. As a result of van der Waals layered materials, the interlayer coupling strength is largest in near-0° and near-60° bilayers, while it is smallest in near-30° bilayers, which agrees well with previous studies [36,45,46]. In this way, Raman measurement results also supports the feasibility of tailoring interlayer coupling by stacking MoS_2_ monolayers into various alignments, as confirmed by PL experimental results in Figure 3.

### 3.2. PL Enhancement in MoS_2_ Bilayers with Anomalous Interlayer Coupling

**Figure 5:**
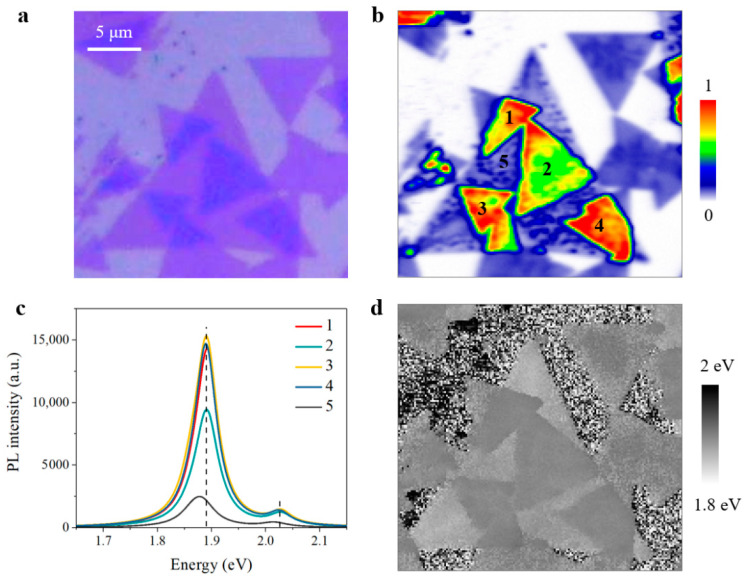
PL mapping of twisted MoS_2_ bilayers with anomalous interlayer coupling. (**a**) Optical image of anomalous bilayers. (**b**) Normalized PL mapping result of anomalous bilayers in (**a**). The PL intensity is integrated within A-exciton resonance. (**c**) PL spectra of monolayer and anomalous bilayer regions marked in (**b**). (**d**) The emission peaks of PL intensity maximum in (**b**).

While most twisted MoS_2_ bilayers demonstrate normal interlayer coupling and resultant lower PL yield in Figure 2, twisted-angle-dependent PL properties in Figure 3, and Raman behaviors in Figure 4 (as mentioned above), unexpected anomalous interlayer coupling effect was observed in other twisted bilayers from the same sample preparation. As can be seen in Figure 5a, there is no difference between anomalous bilayers and normal bilayers from the optical image, and both appear to consist of two stacked monolayers. We thus performed PL mapping measurements to study the optical properties of anomalous bilayers underlying the morphological similarity. Interestingly, giant PL enhancement can be observed in these anomalous bilayers in Figure 5b, instead of the expected PL decrement in normal bilayers as shown in Figure 2. For a better understanding, we collected PL spectra of monolayer and four anomalous bilayers with different twisted angles for comparison, as shown in Figure 5c. As compared with monolayer region 5, PL enhancement is about 4-fold in anomalous bilayer region 2 and even reaches 6-fold in region 1, region 3, and region 4. Apart from the giant PL enhancement, emission peaks of both A-exciton and B-exciton are evidently blueshifted in these anomalous bilayers. The emission peaks of PL intensity maximum are extracted and collected in Figure 5d, unambiguously revealing the blueshifted emission property of the anomalous bilayers. It is worth noting that the twisted angles of bilayer region 1, region 3, and region 4 are different, while PL spectra of these three anomalous bilayers are nearly identical in Figure 5c. This contrasting phenomenon indicates that anomalous interlayer coupling is barely dependent of twisted angle, which results in quite unique emission behaviors instead of the twisted-angle-modulated optical properties observed in normal bilayers.

**Figure 6:**
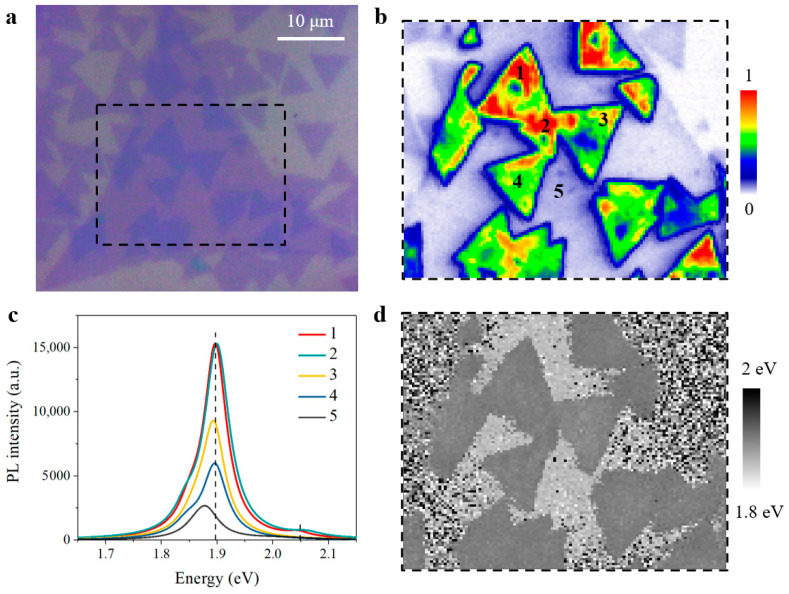
PL mapping of twisted MoS_2_ bilayers with anomalous interlayer coupling from the second sample batch. (**a**) Optical image. (**b**) Normalized PL mapping result. (**c**) PL spectra of monolayer and anomalous bilayer regions marked in (**b**). (**d**) The emission peaks of PL intensity maximum in (**b**).

We firstly considered that one possible reason for unique emission behavior in anomalous bilayers may refer to the transfer processes during sample preparation. Sequential two-step transfer processes would potentially bring in defect, trap, or boundary state between the top and bottom layers. However, these factors give rise to non-radiative pathways and result in PL decrement [5,47], instead of giant PL enhancement as observed in anomalous bilayers. To verify the repeatability of anomalous MoS_2_ bilayers and accompanying unique emission behavior, we followed the same sample preparation method and fabricated the second batch of twisted MoS_2_ bilayers for comparison. Interestingly, most twisted bilayers in the second sample batch exhibited normal interlayer coupling, which was expected and discussed above. Again, we still observed the anomalous emission behavior in other bilayers in the second sample batch. As shown in Figure 6, the PL mapping results of the second sample batch distinctly demonstrate the unique emission behavior from anomalous bilayers, including giant PL enhancement and evidently blueshifted emission peaks. These consistent experimental results and phenomena in Figure 5 and Figure 6 indeed confirm the anomalous interlayer coupling in twisted MoS_2_ bilayers.

**Figure 7:**
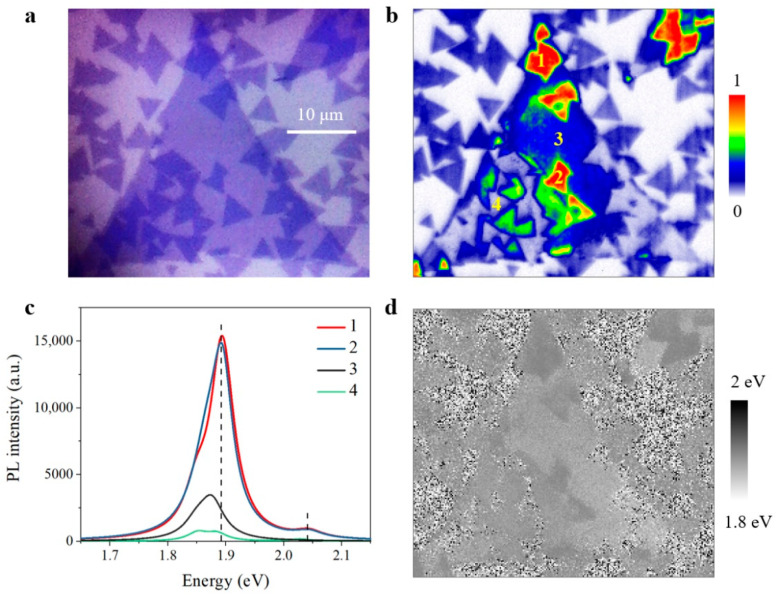
PL mapping of both normal and anomalous twisted MoS_2_ bilayers on top of a same bottom monolayer. (**a**) Optical image. (**b**) Normalized PL mapping result in (**a**). (**c**) PL spectra of monolayer, normal bilayer and anomalous bilayer regions marked in (**b**). (**d**) The emission peaks of PL intensity maximum in (**b**).

With research continuing, we even discovered that the normal and anomalous interlayer coupling can coexist in few sample regions. As can be seen in Figure 7, normal and anomalous twisted MoS_2_ bilayers are both observed on top of the same bottom material. According to the experimental results from Figure 7, the observation of separately distributed normal and anomalous bilayers again indicates that the twisted angle has little effect on unique emission behavior in anomalous bilayers. The underlying origin of anomalous interlayer coupling will be discussed later.

**Figure 8:**
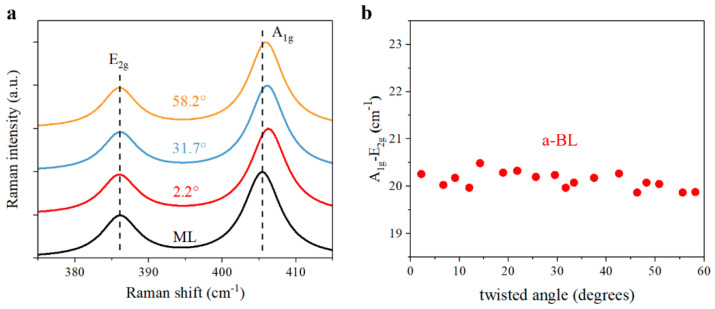
Raman spectra of MoS_2_ bilayers with anomalous interlayer coupling. (**a**) Raman signals of $A_{1g}$ and $E_{2g}$ modes in prototypical monolayer and anomalous bilayers. ML, monolayer. (**b**) Raman interval of $A_{1g}$ and $E_{2g}$ modes. a-BL, anomalous bilayer.

We carried out Raman experiments to further explore the optical properties in twisted MoS_2_ bilayers with anomalous interlayer coupling. Compared with monolayer MoS_2_, the in-plane $E_{2g}$ mode nearly remains unchanged, while the out-of-plane $A_{1g}$ mode is blueshifted slightly in Figure 8a. Unlike the twisted-angle-dependent Raman interval of normal bilayers in Figure 4, the variation of Raman interval between $A_{1g}$ and $E_{2g}$ peaks is rather slight in different anomalous bilayers. In addition, the Raman interval of anomalous bilayers, about 20 cm^−1^ wavenumber, is smaller than that of normal bilayer, which generally ranges from 21 cm^−1^ to 23 cm^−1^ wavenumber. Since the Raman interval reflects the microcosmic interlayer distance, the smaller Raman interval clearly implies a larger interlayer distance and weaker interlayer coupling strength in anomalous bilayers. According to previous reports [30,31], weaker interlayer coupling strength can help increase PL yield of bilayer and many-layer MoS_2_ owing to the modification of band structure. As interlayer distance increasing and the indirect transition being suppressed, bilayer and many-layer can also exhibit direct-bandgap-like emission, which is quite similar to our anomalous bilayers with giant PL intensity enhancement. In this way, the top layer and bottom layer are weakly coupled and the top layer material can be potentially suspended in anomalous bilayers.

**Figure 9:**
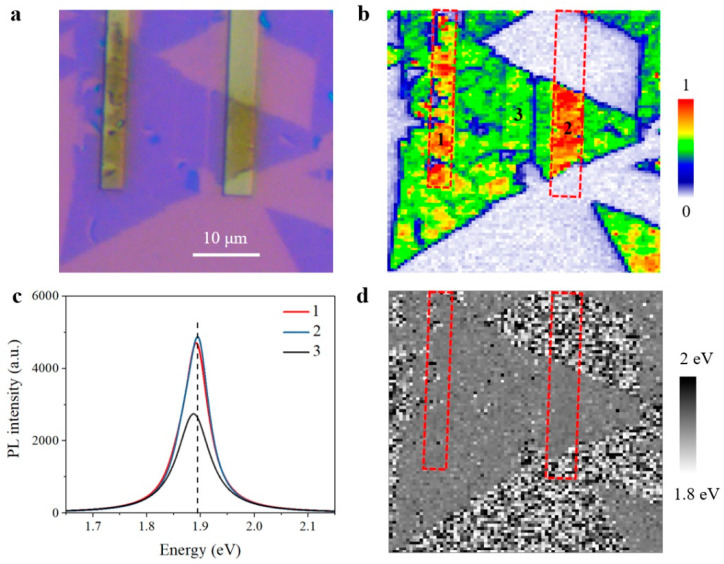
PL mapping of suspended monolayer MoS_2_. (**a**) Optical image. Yellow regions: etched trench. (**b**) Normalized PL mapping result of (**a**). Red dashed boxes: etched trench in (**a**). (**c**) PL spectra of regions marked in (**b**). (**d**) Emission peaks of PL intensity maximum in (**b**).

To estimate the relationship between potentially suspended state of top layer material and PL enhancement in anomalous bilayers, we transferred monolayer MoS_2_ onto SiO_2_ substrate with trench structure fabricated by reactive ion etching and then conducted the PL mapping experiment, which is illustrated in Figure 9. As can be seen, only monolayer MoS_2_ on trench is suspended, and the other part is supported by SiO_2_ substrate for comparison. In Figure 9b, the suspended region of monolayer MoS_2_ corresponds to higher PL intensity, compared with the supported region. Corresponding PL spectra are presented in Figure 9c, where about two-fold PL enhancement is achieved in suspended region. Additionally, emission peaks in suspended area are similarly blueshifted to some extent. Considering the similarity between emission properties of suspended monolayer MoS_2_ and anomalous bilayers, suspension of the top layer material can be regarded as one convincing factor that contributes to anomalous interlayer coupling and resultant PL and Raman properties, as observed from Figure 5, Figure 6, Figure 7 and Figure 8.

### 3.3. Carrier Dynamics in MoS_2_ Bilayers with Anomalous Interlayer Coupling

**Figure 10:**
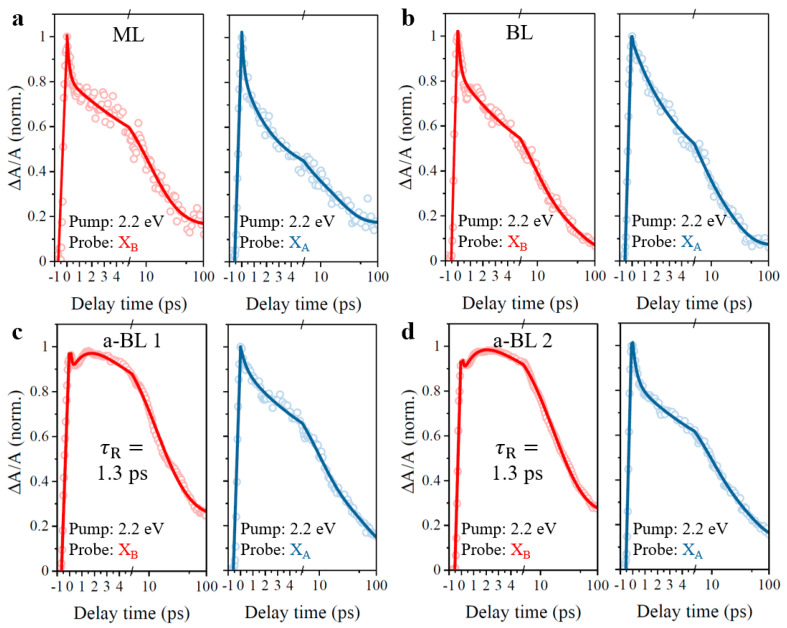
Pump-probe dynamics with off-resonant 2.2 eV excitation. (**a**) ML, monolayer MoS_2_. (**b**) BL, normal bilayer. (**c**) a-BL 1, anomalous bilayer 1. (**d**) a-BL 2, anomalous bilayer 2. Samples from (**a**) to (**d**) are in accordance with region 3, region 4, region 1, and region 2 in Figure 7. X_B_, B-exciton resonance; X_A_, A-exciton resonance.

Since the optical properties are quite different in normal and anomalous bilayers, carrier dynamics with anomalous interlayer coupling requires further study. For better comparison, carrier dynamics with contrasting interlayer coupling were explored with pump-probe measurements in monolayer, normal, and anomalous bilayer regions marked in Figure 7, respectively. Limited by the experimental conditions, the pump and probe beams were both linearly polarized. For comparison, the recorded transient absorption signals were normalized for both A-exciton and B-exciton resonances. Firstly, we performed pump-probe experiments with off-resonance 2.2 eV excitation and related results are presented in Figure 10. As can be seen, carrier dynamics of both A-exciton and B-exciton resonances show similar traces in the monolayer and normal bilayer samples, as well as A-exciton resonance in anomalous bilayers, where transient absorption signals decrease monotonously after the initial rising edge. These transient absorption signals can be fitted with common exponential decay model [48,49], which is described as ΔA(t)/A=A1e−tτ1+A2e−tτ2+A3e−tτ3+A0. In this model, the ΔA(t)/A, $A_{i\;}\;$ and $\tau_{i\;}$ are dynamical transient absorption signal, decay ratio, and corresponding recombination lifetime, $A_{0}$, is a fitting constant. However, the pump-probe signal of B-exciton resonance in anomalous bilayers is no longer monotonously degressive and thus cannot follow common exponential decay model, since the secondary rising behavior of transient absorption signal can be observed in the first few picoseconds after the initial rising edge. To analyze the secondary rising behavior, the recombination model of B-exciton resonance in anomalous bilayers is then corrected with additional item and described as ΔA(t)/A=A1e−tτ1+A2e−tτ2+A3e−tτ3+AR(1−e−tτR), where $A_{R\;}$ and $\tau_{R\;}$ refer to the ratio and lifetime of secondary rising behavior, respectively. By fitting the dynamical recombination curves, the decay parameters of Figure 10 are summarized in Table 1. Interestingly, under off-resonant 2.2 eV excitation, the secondary rising lifetimes of B-exciton resonance in anomalous bilayers are identical, both referring to 1.3 ps for anomalous bilayer 1 and anomalous bilayer 2, respectively.

**Figure 11:**
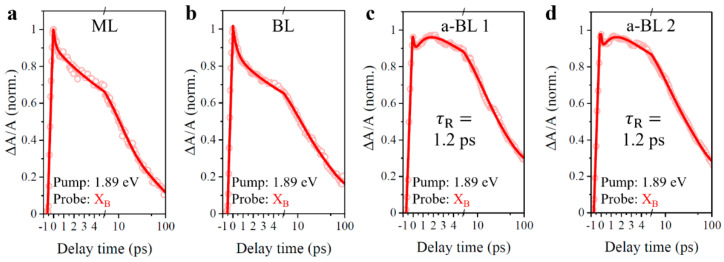
Pump-probe dynamics at B-exciton resonance with 1.89 eV excitation of A-exciton resonance. (**a**) ML, monolayer MoS_2_. (**b**) BL, normal bilayer. (**c**) a-BL 1, anomalous bilayer 1. (**d**) a-BL 2, anomalous bilayer 2. Samples from (**a**) to (**d**) are in accordance with region 3, region 4, region 1, and region 2 in Figure 7. X_B_, B-exciton resonance.

For deeply recognizing the origin of secondary rising behavior of B-exciton resonance in anomalous bilayers, we conducted the pump-probe experiments again with 1.89 eV excitation of A-exciton resonance. In this case, the pump laser, overlapping the transient absorption spectrum within A-exciton resonance, was then filtered out, as well as spectrum signals within A-exciton resonance around 1.89 eV. Transient absorption signals of B-exciton resonance were recorded, as presented in Figure 11. Under 1.89 eV excitation of A-exciton resonance, the transient absorption signals of B-exciton resonance in the monolayer and normal bilayer samples show monotonously degressive trends, similar to results in Figure 10. Surprisingly, the secondary rising behaviors of B-exciton resonance persist in anomalous bilayers, and the extracted rising lifetimes are 1.2 ps by fitting the decay curves, with corresponding fitting results of Figure 11 summarized in Table 2. Again, these consistent results of secondary rising lifetimes verify the aforementioned inference that the anomalous interlayer coupling effect is barely twisted-angle-dependent.

**Figure 12:**
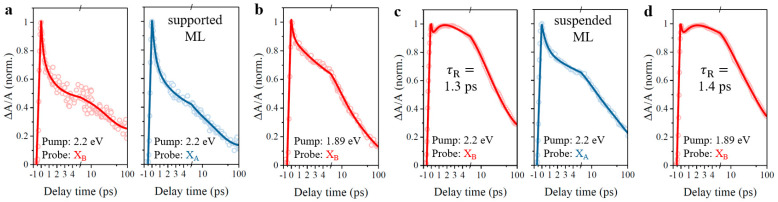
Pump-probe dynamics of supported and suspended monolayer MoS_2_. (**a**,**b**) Supported monolayer MoS_2_. (**c**,**d**) Suspended monolayer MoS_2_. ML, monolayer. X_B_, B-exciton resonance. X_A_, A-exciton resonance.

For comparison, we repeated the pump-probe experiments successively with off-resonant 2.2 eV and 1.89 eV excitation of A-exciton resonance for supported and suspended monolayer MoS_2_, aforementioned in Figure 9, and the corresponding dynamical results are illustrated in Figure 12. As expected, the transient absorption signals of A-exciton and B-exciton resonance in the supported monolayer, as well as A-exciton resonance in the suspended monolayer, decay monotonously in Figure 12. As for B-exciton resonance in the suspended monolayer, we observed secondary rising behaviors in corresponding transient absorption signals in Figure 12, with 1.3 ps and 1.4 ps secondary rising lifetime under off-resonant and resonant excitation conditions, respectively. Corresponding fitting parameters of Figure 12 are summarized in Table 3. According to the observed similarities in emission properties and carrier dynamics between the anomalous bilayers and suspended monolayer, suspension of top monolayer material is reasonably proved as one crucial factor that contributes to anomalous interlayer coupling, resulting in the anomalous giant PL enhancement and dynamical secondary rising behavior in pump-probe results.

Now we turn to discuss the origin of observed dynamical secondary rising behavior of B-exciton resonance, which may be complex owing to strong exciton-exciton [50,51] and exciton-phonon interactions [52,53] in two-dimensional TMDCs. Considering the increased interlayer distance in anomalous bilayers and giant valence band splitting in MoS_2_ [54], the carriers that give rise to pump-probe signals of B-exciton resonance can refer to B-exciton, conduction band electron, or both of them under 1.89 eV excitation of A-exciton resonance. We firstly considered the mechanisms that result in the actual B-exciton under excitation of the A-exciton resonance, including two-photon-absorption [55], Dexter-like intervalley coupling [56], and intravalley exchange [57] of A-exciton and B-exciton. Generally, the resonant 1.89 eV excitation of A-exciton resonance, much lower than the transition bandgap of B-exciton, cannot directly generate B-exciton in MoS_2_ materials through the common band-edge absorption mechanism. However, with increased excitation fluence of A-exciton resonance, B-exciton can be generated by two-photon-absorption process and then emit upconverted PL, as reported previously [55]. Compared with two-photon-absorption process, the intervalley and intravalley exchange mechanisms are relatively complex. For the former Dexter-like intervalley coupling mechanism, a cascade conversion from A-exciton to B-exciton completes with the help of intermediary excited A-exciton, as theoretically and experimentally demonstrated by Berghäuser et al. [56]. After resonant excitation of A-exciton in K valley, the initially generated 1*s* state firstly couples to higher excitonic states with same spin configuration in K valley through inter-exciton coupling. Meanwhile, the intervalley Dexter-like coupling gives rise to an intervalley oscillation transfer from excited A-exciton in K valley to B-exciton in opposite -K valley, which finally generates B-exciton population without directly pumping the band-edge transition of B-exciton. As for the latter intravalley excitonic exchange reported by Guo el al. [57], they claim that the interaction between electron and hole consists of a direct screened Coulomb interaction and an additional exchange bare Coulomb interaction. The repulsive exchange interaction involving the bare Coulomb interaction leads to exciton eigenstates consisting of electron-hole states with mixed spins, resulting in excitonic states that are no longer Ising excitons. According to their theoretical calculation, the 1*s* state of actual A-exciton primarily includes original Ising A-exciton, but also mixes in 3.6% of original Ising B-exciton by the intravalley exchange interaction. In this way, B-exciton can be generated and resultant PL signals are observed within an ultrafast timescale upon excitation of A-exciton resonance.

**Figure 13:**
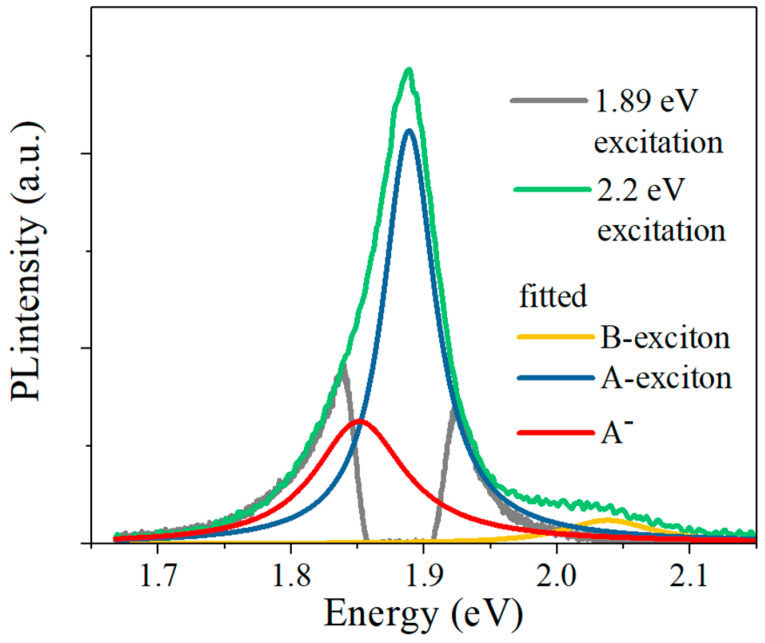
PL spectra of anomalous MoS_2_ bilayer with off-resonant and resonant excitation. PL spectrum with off-resonant 2.2 eV excitation is rescaled and fitted for direct comparison. No emission of B-exciton can be observed under 1.89 eV excitation of A-exciton resonance. A^-^, trion.

We noticed that these three mechanisms all refer to actual B-exciton and resultant PL phenomenon. Therefore, we conducted a contrastive PL experiment in anomalous bilayer sample under off-resonant and resonant excitation conditions, as presented in Figure 13. Under resonant 1.89 eV excitation condition, a notch filter (NF658-26, Thorlabs) was utilized to eliminate the pump laser, making the corresponding PL spectrum inconsecutive and unable to be fitted. For comparison, the PL spectrum of 2.2 eV excitation was rescaled to fit the PL spectrum lineshape of 1.89 eV excitation. As can be seen in Figure 13, the PL spectrum consists of emissions from trion, A-exciton, and B-exciton under off-resonant 2.2 eV excitation. However, no emission of B-exciton can be observed under resonant 1.89 eV excitation, which indicates that no B-exciton is actually generated in such condition, thus excluding the two-photon-absorption, Dexter-like intervalley coupling, and intravalley exchange in the anomalous bilayer sample.

Since the contrastive PL experiment in Figure 13 rules out the mechanisms of actual B-exciton under excitation of A-exciton resonance, transient absorption signal of B-exciton resonance, as well as the secondary rising behavior, is then convincingly assigned to conduction band electron. As reported previously [58], both intravalley spin flip and intervalley scattering of the conduction band electron in A-exciton were proved to give rise to pump-probe signals of B-exciton resonance. Unlike giant valence band splitting energy, the conduction band splitting energy is rather small in TMDCs, enabling the intravalley spin flip of conduction band electron. Owing to only 3 meV conduction band splitting in MoS_2_ [54,59], it was reported that the pump-probe signal of B-exciton resonance can arise simultaneously within the rising edge of A-exciton signal upon resonant excitation in the same valley, revealing an ultrafast intravalley spin flip of conduction band electron within 100 fs at 77 K [58]. Considering that the phonon population increases with temperature, the intravalley spin flip of conduction band electron can be theoretically faster at room temperature. As for intervalley scattering of conduction band electron, Wang et al. also proved that it occurs within an ultrafast timescale similar to that of intravalley spin flip [58], which is far smaller than the observed 1.2 ps secondary rising lifetime of pump-probe signal of B-exciton resonance in Figure 11. In this way, both intravalley spin flip and intervalley scattering of conduction band electron are potentially related to the initial rising edge of pump-probe signals of B-exciton resonance, rather than the anomalous secondary rising behavior.

**Figure 14:**
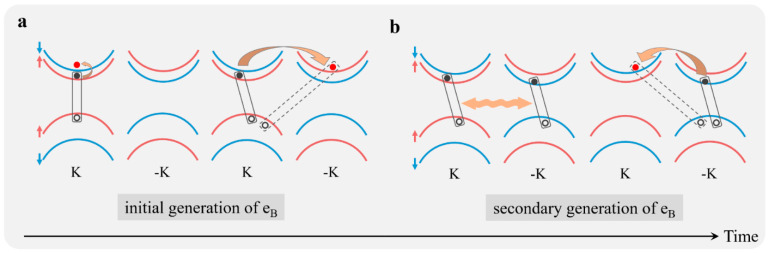
Phenomenological illustration of anomalous pump-probe signal of B-exciton resonance with excitation at A-exciton resonance. (**a**) Intravalley spin flip and direct exciton-phonon scattering give rise to the initial generation of pump-probe signal. (**b**) The secondary rising of pump-probe signal at B-exciton resonance results from intervalley exchange of A-exciton within valley lifetime and subsequent exciton-phonon scattering. Red (blue) arrows: spin up (down). e_B_, conduction band electron contributing to pump-probe signal of B-exciton resonance, marked in red.

It is worth noting that the observed secondary rising lifetime of pump-probe signals of B-exciton resonance is very consistent with the valley lifetime on 1 ps order in TMDCs at room temperature [60,61,62,63], which implies a relationship between the pump-probe signal and valley depolarization mechanisms. As theoretically and experimentally demonstrated by Selig et al. [64], they deduced the pump-probe signal with interplay of dark exciton, intervalley exchange and phonon, and discovered that corresponding pump-probe signal of B-exciton resonance rises much slower with prolonged 0.7 ps rising edge upon 20 fs resonant excitation of A-exciton, which agrees well with our 1.2 ps secondary rising lifetime in the same order of magnitude. Based on our experimental results, we thus introduced a reasonable phenomenological model to explain observed pump-probe signal of B-exciton resonance, including the initial generation and secondary rising behavior with excitation of A-exciton resonance, which is illustrated in Figure 14. For the convenience of discussion, the resonant pump laser is simplified to left-circularly polarized component, having no effect on the conclusions and, thus, only A-exciton can be optically generated in K valley upon excitation. Firstly, as intravalley spin flip (direct exciton-phonon scattering) occurs within an ultrafast timescale in Figure 14a, the conduction band electron of A-exciton transits to the upper conduction band in the same (opposite) valley with inverted (conserved) spin, resulting in the initial rising edge of pump-probe signal of B-exciton resonance. Afterwards in Figure 14b, the intervalley exchange induces A-exciton population in -K valley within the valley lifetime. Electron of A-exciton in -K valley subsequently transits back to the upper conduction band in K valley through exciton-phonon scattering, finally giving rise to the secondary rising behavior in pump-probe signal of B-exciton resonance. Considering the fact that the probe beam is linear polarized, pump-probe signals actually consists of dynamical responses from both K and -K valleys. Therefore, this combined phenomenological model accords closely with measured dynamical results in the time domain, as observed in Figure 11c,d.

**Figure 15:**
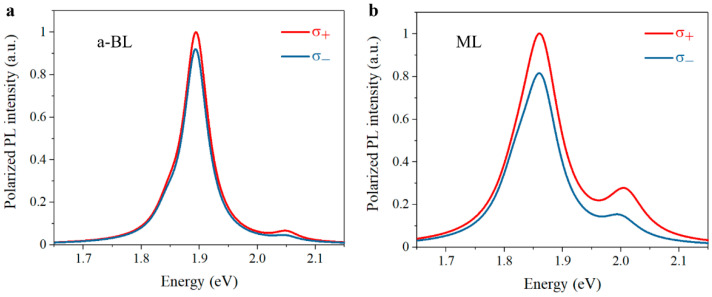
Normalized polarization-resolved PL spectra. (**a**) a-BL, anomalous bilayer. (**b**) ML, monolayer. The 2.2 eV excitation laser is left-circularly (σ_+_) polarized. Co-polarized and cross-polarized PL components are marked in red and blue, respectively.

Considering that the pump-probe signal of B-exciton resonance is related to valley depolarization of A-exciton in the phenomenological model, the evident secondary rising behavior thus indicates stronger intervalley exchange and exciton-phonon scattering, which implies potentially degraded valley polarization in anomalous bilayer sample. In this way, we finally carried out polarization-resolved PL measurements to estimate the valley polarization in the anomalous bilayer sample and the corresponding results are presented in Figure 15. As expected, evident degradation of valley polarization is observed in the anomalous bilayer compared with that of monolayer sample, which verifies the validity of the established phenomenological model in Figure 14.

## 4. Conclusions

In summary, we explored optical properties and carrier dynamics in artificially twisted MoS_2_ bilayers. While most twisted bilayers showed twisted-angle-dependent optical properties, anomalous optical properties were firstly observed in other MoS_2_ bilayers, including giant PL enhancement and blueshifted emission peaks that were independent of twisted angles. By comparing our results with the emission properties of suspended monolayer sample, the anomalous PL enhancement in MoS_2_ bilayers was found to be related to suspension of the top layer material. Carrier dynamics in anomalous MoS_2_ bilayers were revealed and analyzed with pump-probe measurements, while the corresponding phenomenological model, based on valley depolarization, was established. Specifically, under excitation of A-exciton resonance, intravalley spin flip and direct exciton-phonon scattering give rise to the initial generation of pump-probe signal of B-exciton resonance. The secondary rising behavior in the pump-probe signal of B-exciton resonance is firstly reported, which originates from intervalley exchange of A-exciton within valley lifetime and subsequent exciton-phonon scattering. These results can help recognize the intrinsic optical properties of TMDCs and shed light on the potential applications beyond TMDCs monolayers.

## Figures and Tables

**Table 1:** Fitted decay parameters for off-resonant 2.2 eV excitation.

Item	ML		BL		a-BL 1		a-BL 2	
	X_B_	X_A_	X_B_	X_A_	X_B_	X_A_	X_B_	X_A_
$\tau_{1}$ (ps)	0.2 (19%)	0.2 (18%)	0.2 (20%)	1.95 (13%)	0.1 (10%)	0.5 (10%)	0.1 (11%)	0.3 (17%)
$\tau_{2}$ (ps)	9.5 (40%)	2.4 (36%)	7.1 (43%)	4.4 (41%)	10 (47%)	8.14 (48%)	11.2 (41%)	7.3 (37%)
$\tau_{3}$ (ps)	26 (25%)	18.8 (31%)	31 (29%)	20.2 (37%)	29.7 (39%)	50.9 (34%)	29.4 (40%)	46 (33%)
$A_{0}$	(16%)	(15%)	(8%)	(9%)		(8%)		(13%)
$\tau_{R}$ (ps)					1.3 (25%)		1.3 (26%)	

**Table 2:** Fitted decay parameters for resonant 1.89 eV excitation.

Item	ML	BL	a-BL 1	a-BL 2
	X_B_	X_B_	X_B_	X_B_
$\tau_{1}$ (ps)	0.2 (10%)	0.4 (13%)	0.2 (12%)	0.1 (10%)
$\tau_{2}$ (ps)	9.6 (44%)	7.5 (35%)	8.5 (42%)	7.4 (39%)
$\tau_{3}$ (ps)	69.6 (32%)	41.8 (39%)	39.2 (46%)	43.2 (49%)
$A_{0}$	(14%)	(13%)		
$\tau_{R}$ (ps)			1.2 (26%)	1.2 (23%)

**Table 3:** Fitted decay parameters for supported and suspended monolayer MoS_2_.

Item	Supported ML (2.2 eV pump)		Suspended ML (2.2 eV pump)		Supported ML (1.89 eV pump)	Suspended ML (1.89 eV pump)
	X_B_	X_A_	X_B_	X_A_	X_B_	X_B_
$\tau_{1}$ (ps)	0.2 (20%)	0.4 (20%)	0.1 (7%)	0.6 (17%)	0.3 (12%)	0.2 (11%)
$\tau_{2}$ (ps)	1.3 (34%)	2.7 (37%)	12 (47%)	8.1 (31%)	7.5 (45%)	7.4 (44%)
$\tau_{3}$ (ps)	26.7 (25%)	24.5 (30%)	64.9 (46%)	64 (38%)	49.3 (35%)	44.9 (45%)
$A_{0}$	(21%)	(13%)		(14%)	(8%)	
$\tau_{R}$ (ps)			1.3 (17%)			1.4 (18%)

## Data Availability

The data presented in this study are available on request from the corresponding author.
